# Analgesia efficacy of erector spinae plane block in laparoscopic abdominal surgeries: a systemic review and meta-analysis

**DOI:** 10.1097/JS9.0000000000001421

**Published:** 2024-04-03

**Authors:** Crystal Jin-Yang Sia, Sheila Wee, Angie Phui-Sze Au-Yong, Sui-An Lie, Winson J. Tan, Fung-Joon Foo, Juinn-Huar Kam, Daniel JK Lee, Frederick H. Koh

**Affiliations:** a Department of Colorectal Surgery, Sengkang General Hospital; b Department of General Surgery, Sengkang General Hospital; c Department of Anaesthesiology and Perioperative Science, Singapore General Hospital; d Department of Anaesthesiology, Khoo Teck Phuat Hospital; e Department of Colorectal Surgery, Khoo Teck Puat Hospital, Singapore

**Keywords:** abdominal surgery, erector spinae plane block, ESP block, ESPB, laparoscopic, laparoscopy

## Abstract

**Background:**

Multimodal analgesia is now widely practised to minimise postoperative opioid consumption while optimising pain control. The aim of this meta-analysis was to assess the analgesic efficacy of erector spinae plane block (ESPB) in patients undergoing laparoscopic abdominal surgeries. This will be determined by perioperative opioid consumption, subjective pain scores, and incidences of postoperative nausea and vomiting.

**Methods:**

The authors systemically searched electronic databases for randomised controlled trials (RCTs) published up to February 2023 comparing ESPB with other adjuvant analgesic techniques in laparoscopic abdominal surgeries. Nine randomised controlled trials encompassing 666 subjects were included in our study.

**Results:**

ESPB was shown to reduce postoperative opioid consumption [mean difference (MD) of −5.95 mg (95% CI: −8.86 to −3.04; *P*<0.0001); *I*^2^=89%], intraoperative opioid consumption MD of −102.4 mcg (95% CI: −145.58 to −59.21; *P*<0.00001); *I*^2^=39%, and incidence of nausea [RR 0.38 (95% CI: 0.25–0.60; *P*<0.0001); *I*^2^=0%] and vomiting [RR 0.32 (95% CI: 0.17–0.63; *P*=0.0009); *I*^2^=0%] in laparoscopic abdominal surgeries. Subgroup analysis on laparoscopic colorectal surgeries further showed reduction in postoperative pain scores MD of −0.68 (95% CI: −0.94 to −0.41); *P*<0.00001; *I*^2^=0%].

**Conclusions:**

This study concludes that ESPB is a valuable technique with proven efficacy to potentially promote faster postoperative recovery through optimising pain control while minimising opioid requirements.

## Introduction

Highlights

- Erector spinae plane block (ESPB) stands out due to its relative simplicity in comparison to other interfascial plane blocks.
- ESPB reduced perioperative opioid consumption after laparoscopic abdominal surgeries thus contributing to the goals of enhanced recovery after surgery.
- Reduction of opioid consumption with the use of ESPB reduces incidences of postoperative complications such as nausea and vomiting.
- ESPB is an effective tool in the postoperative analgesic armamentarium.

Enhanced Recovery after Surgery (ERAS) protocols are rapidly becoming the standard of care for patients undergoing elective surgery with aims to expedite postoperative recovery and decrease morbidity with multimodal analgesia^1^. This encompasses not only oral analgesics like NSAIDS and opioids but also patient-controlled analgesia (PCA), and locoregional anaesthetic techniques such as epidural analgesia or interfascial plane blocks^1^.

Interfascial plane blocks (IPB) first came into the limelight with the introduction of the transversus abdominis plane block (TAPB) technique in 2001. This inspired a flurry of practise-changing research, giving rise to other variations such as the subcostal TAP, pectoralis, transversalis fascia, and erector spinae plane block (ESPB) among others.

ESPB was first demonstrated by Forero *et al*.^2^ in 2016 in two case studies to primarily address thoracic neuropathic pain in metastatic and malunion of rib fractures. Many other studies have since been performed to evaluate its analgesic efficacy in abdominal^3–5^ and thoracic^6,7^ surgeries. ESPB aims to provide both somatic and visceral analgesia although its mechanism of action has yet to be fully elucidated^8^. MRI studies proposed that infiltration of anaesthetic medications into the plane beneath the erector spinae results in both transforaminal and epidural spread. A block at the foramen where the thoracolumbar splanchnic nerves emerge results in visceral pathway blockade while epidural spread facilitates segmental blockade, addressing somatic pain^9^. The erector spinae muscle extends within the paravertebral groove on either sides of the vertebrae column, running from the skull to sacrum while being encased within a single aponeurosis. As such, local anaesthetic agents injected into this sub-fascial plane can spread in a cranio-caudal manner. Cadaveric studies have documented the extent of spread to be about three to four levels cranially and caudally^10^. When injected at the lower thoracic region, ESPB affects the lower thoracic nerve roots supplying the abdomen and hence, can potentially be used to provide analgesia for abdominal surgeries.

ESPB stands out amongst other neuroaxial blocks because of its relative simplicity, ability to effectively provide both somatic and visceral analgesia to a wide area of the abdomen with a single level injection and optimal safety profile. The sonoanatomy of ESPB is easy to recognise and the anaesthetic agent is infiltrated within a plane far from crucial structures such as the pleura, vessels and medulla^11^. This is unlike the other blocks such as the thoracic paravertebral plane block (TPVB), which can reliably achieve both visceral and somatic^12^ analgesia, but the benefits must be weighed against its relative complexity and complications such as pneumothorax^13^. In the case of epidural analgesia, its implementation has been associated with increased hemodynamic instability due to the sympatholytic effect of neuraxial blockade^14^. Transient detrusor muscle dysfunction caused by epidurals also necessitate the use of indwelling bladder catheters which increases the risk of catheter-associated urinary tract infection (CAUTI)^15^.

Despite its benefits, widespread adoption of ESPB has yet to gain significant traction.

This study aims to systematically review the literature reporting the efficacy of the ESPB as an adjuvant analgesia in laparoscopic surgeries. The primary outcome measure would be postoperative opioid consumption. Secondary outcomes are: intraoperative opioid consumption, postoperative pain scores and incidences of nausea and vomiting.

## Methods

### Literature review and search strategy

This review was conducted in line with the PRISMA^16^ (Preferred Reporting Items for Systemic Reviews and Meta-Analysis) and AMSTAR^17^ (Assessing the methodological quality of systemic review) guidelines. The scope of the review included randomised controlled trials (RCT) reporting the use of ESPB in laparoscopic abdominal surgeries in adults. Embase, PubMed, Scopus, and Google Scholar were searched up to February 2023. The search strategy and analytic plan of this systematic review and meta-analysis has been registered on PROSPERO.

### Criteria for review

**Table:** 

Inclusion criteria	Exclusion criteria
Randomised controlled trials Single dose ultrasound guided ESPB administration Papers published in English or translated to English Adults aged ≥18years Reported outcome of postoperative opioid consumption within 24 h	Surgeries suitable for day-case and short-stay units, that is, Laparoscopic cholecystectomy, Laparoscopic appendicectomy, Laparoscopic cystectomy Open surgery Case reports, Study protocol, Conference abstracts Papers not in English language

Procedures such as laparoscopic appendicectomy, cholecystectomy^18^, and ovarian cystectomies are now increasingly performed as day or short stay unit procedures. Postoperative pain can routinely be well controlled with regular NSAIDs, Paracetamol, and opioid as rescue analgesia. Hence, the use of interfascial plane blocks in patients undergoing these subset of procedures may not be indicated especially since the length of stay is often less than 24 h.

### Type of outcome measures

We calculated postoperative opioid consumptions in terms of morphine (mg) using opioid dose conversions: 10 mg of morphine was equivalent to 0.01 mg Sufatenil and 0.1 mg Fentanyl^19^. Secondary outcomes were: intraoperative opioid consumption measured in intravenous fentanyl equivalents (mcg), pain scores quantified by the Visual Analogue Scale (VAS), and incidences of postoperative nausea and vomiting. Four RCTs utilised the Numerical Rating Scale (NRS) to quantify postoperative pain; however, data was not provided within the manuscript to allow for reliable analysis. NRS was therefore not evaluated as a secondary outcome.

### Data extraction and quality assessment

**Figure 1:**
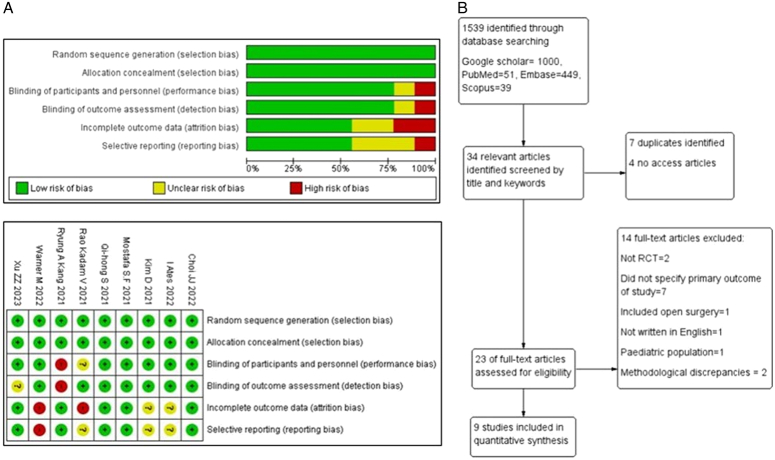
A. Risk of bias assessment of included studies. B. PRISMA flowchart of included and excluded studies.

The data was collected in accordance to the above inclusion and exclusion criteria. The search terms included a combination of ‘erector spinae plane block’, ‘ESP block’, ‘ESPB’, ‘Laparoscopy’, ‘Laparoscopic’, ‘abdominal surgery’, ‘randomised controlled trial’. In papers with limited access, the respective authors were contacted and excluded if the request was unanswered. Quality of the studies was evaluated using the Cochrane Risk of Bias Tool. The risk of bias was graded accordingly into three levels (low risk, unclear risk, and high risk) as seen in Figure 1A.

### Statistical analysis

The continuous variables were expressed as means± SD and categorical variables were presented as proportions. For categorical outcomes, the risk ratio (RR) with 95% CI were calculated. For continuous outcomes, mean difference (MD) with 95% CI were calculated. If median [interquartile range, IQR] data was reported, it was converted into means±SD by adopting the method of Hozo *et al*.^20^. Statistical heterogeneity was estimated by *I*^2^ where *I*^2^ of >50% is considered significant heterogeneity and the random-effects model would be used. *P*-value<0.05 was considered statistically significant. Sensitivity analysis was performed using bootstrapping approach to evaluate whether the results were influenced significantly by a single study. Data analysis were carried out using the Review Manager, Version 5.4.1 while sensitivity analysis was performed using Comprehensive Meta-Analysis, CMA Version 4.

## Results

### Results of literature search

Our study selection process is shown in the PRISMA flow diagram (Fig. 1B). The literature search initially retrieved 1539 citations (Google Scholar 1000, PubMed 51, EmBase 449, Scopus 39). Of which, 34 relevant articles were identified through abstract screening. Nine studies with 666 participants were eventually included in the meta-analysis.

### Patient characteristics and block techniques

Patient characteristics and block techniques are summarised in Table 1. There was no significant difference in baseline characteristic between the ESPB and Non-EPSB groups (which consisted of sham block, no block, intrathecal morphine, TAPB). The ESPB block is performed via ultrasound guidance at various thoracic levels predetermined by the anaesthesiologist involved in the studies. All studies used single shot ropivacaine or bupivacaine at differing concentrations (Min-Max: Ropivacaine 0.25–0.5%, Bupivacaine 0.125–0.25%).

**Table 1:** Patient characteristics and block techniques employed in the included studies.

Author	Study type	Year of publication	Study period	Country	Operative procedure	Total Sample size	ASA	Intervention	Sample size (n)	Block technique	Mean age
Choi JJ *et al.* ^21^	RCT	2021	(not specified)	Incheon, Korea	Laparoscopic colorectal surgery	59	III–IV	ESPB	29	Bilateral; USG; 20 ml 0.5% ropivacaine at T7	58.6±7.1
								Sham block	30	Bilateral; USG; 20 ml normal saline at T7	59.5±7.9
I Ates *et al.* ^22^	RCT	2022	(not specified)	Erzurum, Turkey	Laparoscopic colorectal resection	48	I–III	ESPB	24	Bilateral; USG; 20 ml 0.25% bupivacaine at T7	57.29±10.27
								Wound infiltration	24	20 ml 0.25% bupivacaine to incision	48.96±14.17
Kim D *et al.* ^23^	RCT	2021	January 2019 to September 2021	Korea	Laparoscopic hepatectomy	70	I–II	ESPB	35	Bilateral ; USG; 20 ml 0.5% ropivacaine at T9	57.8±10
								Sham block	35	Bilateral; USG; 20 ml 0.9% saline at T9	56.6±10
Mostafa S F *et al.* ^24^	RCT	2021	August 2017 to September 2018	Tanta, Egypt	Laparoscopic bariatric surgery	60	III	ESPB	30	Bilateral; USG; 20 ml 0.25% bupivacaine at T7	38.80±6.65
								Sham block	30	Bilateral ; USG; 20 ml normal saline at T7	40.30±7.86
Qi-Hong S *et al.* ^25^	RCT	2021	June 2020 to July 2021	China	Laparoscopic colorectal surgery	62	I–III	ESPB	31	Bilateral; USG; 20 ml 0.25% ropivacaine at T9	71.64 ± 4.5
								TAPB	31	Bilateral; USG; 20 ml 0.25% ropivacaine	72.31 ± 6.3
Rao Kadam V *et al.* ^26^	RCT	2021	February 2019 to February 2020	Adelaide, Australia	Laparoscopic colorectal surgery	67	I–III	ESPB	33	Bilateral; USG; 20 ml 0.5% ropivacaine at T8	60.5±17.8
								Wound infiltration	34	40 ml 0.5% ropivacaine to incision	61.2±13.3
Ryung A Kang *et al.* ^27^	RCT	2021	October 2019 and September 2020	Seoul, Korea	Laparoscopic hepatectomy	59	I–II	ESPB	30	Bilateral; USG; 20 ml 0.375% ropivacaine with 5 μg·ml−1 epinephrine at T8	38.6±13.1
								Spinal	29	USG; 0.4 ml 1 mg/ml morphine sulfate at L3/4 or L4/5	37.4±12.1
Warner M *et al.* ^28^	RCT	2022	March 2019 to March 2021	Indiana, USA	Laparoscopic hysterectomy	75	I–IV	ESPB	39	Bilateral; USG; Exparel 10 ml + 30 ml 0.125% bupivacaine at T12	47.1±1.9
								TAPB	36	Bilateral; USG; Exparel 10 ml + 30 ml 0.125% bupivacaine at T12	42.7±1.8
Xu ZZ *et al.* ^29^	RCT	2023	(not specified)	BeiJing, China	Laparoscopic nephroureterectomy	166	I–III	ESPB	83	Bilateral; USG 0.4 ml/kg 0.375% ropivacaine at T10	66±2.31
								Paravertebral block	83	Bilateral; USGl 0.4 ml/kg 0.375% ropivacaine at T10	63.5±3.46

### Outcome analysis

**Figure 2:**
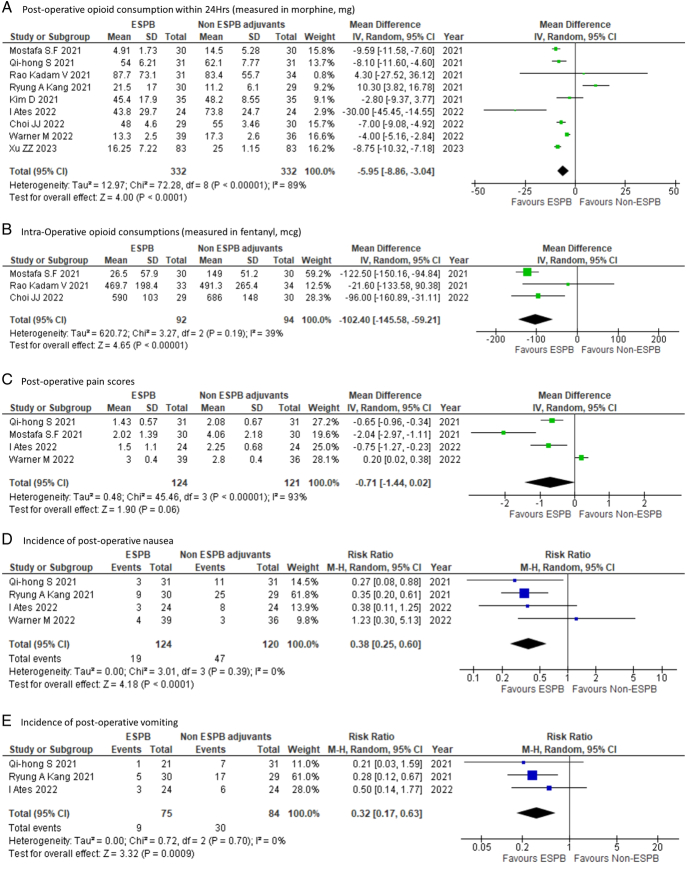
Forest plot of (A) 24 hour cumulative postoperative intravenous morphine consumption in mg (B) intraoperative intravenous fentanyl consumption in mcg (C) pain score at 24 h postoperatively (D) incidence of postoperative nausea in first 24 h (E) incidence of postoperative vomiting in first 24 h; ESPB: erector spinae plane block, df: degree of freedom.

The pooled effects of the nine RCTs examining postoperative opioid consumption within the first 24 h revealed that ESPB significantly reduces the opioid demand as compared to non-ESPB groups MD of −5.95 mg (95% CI: −8.86 to −3.04; *P*<0.0001) but with high heterogeneity (*I*^2^=89%) (Fig. 2A). Similarly, patients that received ESPB had a lower intraoperative opioid requirement MD of −102.4 mcg (95% CI: −145.58 to −59.21; *P*<0.00001; *I*^2^=39%) (Fig. 2B). In terms of postoperative complications, ESPB was further associated with a lower risk of developing postoperative nausea [RR 0.38 (95% CI: 0.25–0.60; *P*<0.0001); *I*^2^=0%] (Fig. 2D) and vomiting [RR 0.32 (95% CI: 0.17–0.63; *P*=0.0009); *I*^2^=0%] (Fig. 2E). ESPB did not significantly reduce postoperative pain scores MD of −0.71 (95% CI: −1.44–0.02; *P*=0.06); *I*^2^=93%] (Fig. 2C).

**Figure 3:**
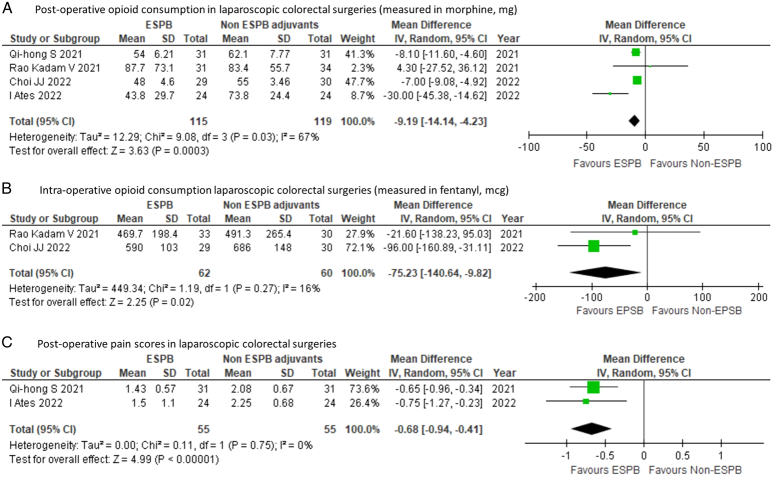
Subgroup analysis of laparoscopic colorectal procedures (A) 24 h cumulative postoperative intravenous morphine equivalent consumption in mg (B) intraoperative intravenous fentanyl equivalent consumption in mcg (C) 24 h postoperative pain scores; ESPB: erector spinae plane block, df: degree of freedom.

Subgroup analysis on laparoscopic colorectal surgeries (four studies, *n*=234) showed that ESPB significantly reduced postoperative opioid MD of −9.19 mg (95% CI: −14.14 to −4.23; *P* =0.0003); *I*^2^=67%] (Fig. 3A) as well as intraoperative opioid consumption MD of −75.23 mcg (95% CI: −140.64 to −9.82); *P*=0.02; *I*^2^=16% (Fig. 3B). Despite the reduction in overall opioid usage, there was also significant reduction in postoperative pain scores MD −0.68 (95% CI: −0.94 to −0.41; *P*<0.00001); *I*^2^=0% (Fig. 3C) observed in patients that received ESPB.

**Figure 4:**
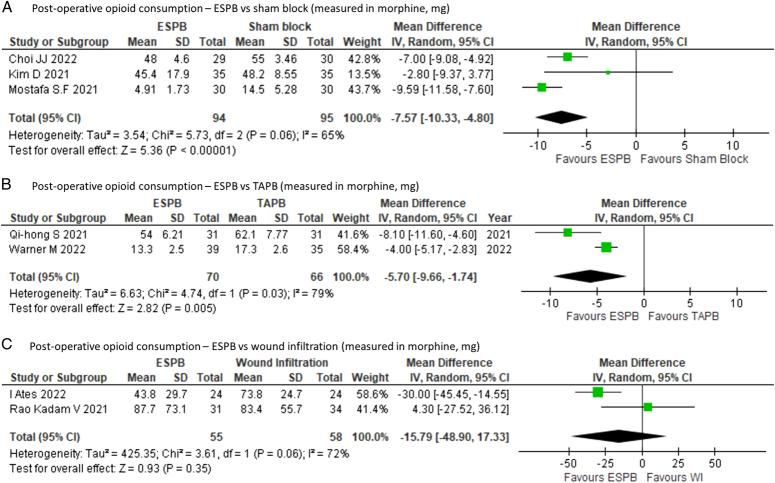
Subgroup analysis comparing postoperative opioid consumption (mg) within 24 h in ESPB vs (A) sham block (B) TAPB (C) wound infiltration; ESPB: erector spinae plane block, df: degree of freedom.

Further subgroup analysis comparing ESPB to sham blocks MD of −7.57 mg (95% CI: −10.33 to −4.80; *P*<0.00001); *I*^2^=65% (Fig. 4A) and TAPB MD of −5.70 mg (95% CI: −9.66 to −1.74; *P*=0.005); *I*^2^=79% (Fig. 4B) demonstrated reduced postoperative opioid consumption. When compared to wound infiltration which was employed in two out of nine studies, ESPB lacked statistical significance in terms of reducing postoperative opioid consumption MD of −15.79 mg (95% CI: −48.90 to 17.33; *P*=0.35); *I*^2^=72% (Fig. 4C).

### Sensitivity analysis

Sensitivity analysis performed using bootstrapping method confirm that the findings were not significantly influenced by any single study (Supporting Information Table 1, Supplemental Digital Content 1,http://links.lww.com/JS9/C322).

## Discussion

No previous study has attempted to assess ESPB in patients undergoing laparoscopic abdominal surgeries as previous studies often included open surgeries, laparotomy and day surgery procedures such as laparoscopic cholecystectomy^3,30^. These are excluded from our analysis which aims to evaluate the use of ESPB in the setting of major laparoscopic abdominal surgeries only. Herein, we focused on the analgesic efficacy of ESPB as compared to other analgesic modalities in this specific population of patients.

This meta-analysis demonstrated that in patients undergoing laparoscopic abdominal surgery such as laparoscopic colorectal or bariatric surgery, the use of ESPB may benefit recovery in terms of reducing perioperative opioid consumption, and incidences of nausea and vomiting. There is; however, no significant difference in the postoperative pain scores when all surgeries were analysed together. Nonetheless, it is worth taking into consideration that while the VAS attempts to objectify pain which is a subjective experience, it is still vulnerable to inter and intraindividual variability^31^. This may be attributed to concomitant physical, physiological, and psychosocial factors that can confound the actual experience of ‘pain’. Reassuringly, subgroup analysis focusing on laparoscopic colorectal surgeries showed that ESPB significantly reduced perioperative opioid requirements while achieving lower postoperative pain scores thus satisfying current-day ERAS recommendations and achieves its intended outcomes.

Therefore, it is reasonable that an effective regional block technique such as the ESPB be incorporated as a standard component of multimodal analgesia for laparoscopic abdominal surgeries.

IPB first came into the picture when Rafi^32^ introduced the TAPB technique in 2001 with the aims of achieving a field block. Advancements in ultrasound technology has allowed for TAPB to become widely employed as it was considered a safe and easy to perform therapeutic adjunct to pain control. However, TAPB can only reliably provide somatic analgesia to the anterior and/or lateral abdominal wall in postoperative patients, significantly limiting its efficacy.

Since its conceptualisation in 2016, ESPB has been unparalleled in terms of popularity in the field of regional anaesthesia. In fact, it has been recently included as one of the seven ‘Plan A’ blocks amongst other established techniques such as the interscalene block for shoulder surgery and the axillary block for upper limb surgery^33^. What sets ESPB apart is its versatility, ease of administration, and favourable safety profile. The ESPB can be performed at all levels of the spine to provide both somatic and visceral analgesia to various regions of the body. Rates of block failures are as low as less 8% when performed by both experienced and nonexperienced anaesthetists^34^ because the sonoanatomy of ESPB is easy to learn, identify, and master. A survey of trainee anaesthetists found that of the seven Plan A blocks, there was a highest confidence level in administrating ESPB^35^. In terms of complications, ESPB is considered a superficial block with low risks for bleeding. In a review of 45 RCTs involving thoracic ESPB by De Cassai *et al*.^36^, there were no reported complications postprocedurally. In a head-on comparison between ESPB and TPVB which has been proven to be one of the most effective regional anaesthesia techniques for postoperative analgesia, ESPB was associated with significantly lower incidences of hemodynamic instability and block failures while offering noninferior analgesic effects^37^. In a similar study comparing ESPB to TPVB after modified radical mastectomy, four out of 35 patients that underwent TPVB developed pneumothorax while none in the ESPB group had similar presentations^38^. ESPB is a simple technique with superficial landmarks, unlike TPVB with close anatomic proximity to pleura and the central neuraxial system.

When pitted against epidural analgesia which has been considered the cornerstone of the ERAS programme especially in the setting of open colorectal surgery^39^, ESPB again demonstrated superiority with fewer procedural complications and noninferior analgesic efficacy^40^. ESPB can also be administered to patients who are coagulopathic or has received recent anticoagulation as it targets the myofascial plane far away from the neuraxial system^41^. Furthermore, epidural analgesia itself has not demonstrated similar success in the setting of laparoscopic abdominal surgeries. A previous meta-analysis performed by Giuseppe *et al*., which looked at five RCTs involving 163 patients found that epidural was in fact associated with a significantly increased duration of stay, without any significant difference in postoperative complications and readmissions as compared to other analgesic techniques^42^. In addition, combining epidural and general anaesthesia may lead to more profound hypotension due to sympathetic blockade and by extension, increase the risk of anastomotic breakdown due to compromised blood supply and oxygen delivery to peripheral tissues during episodes of hypotension. Notwithstanding the hemodynamic implications of epidural analgesia, the placement of epidural catheters in itself may also be challenging with failure rates ranging from 13 to 32%^43^.

This pooled analysis validates the efficacy of ESPB as an adjuvant analgesia in laparoscopic surgeries. Along with its favourable side effect profile and ease of administration, ESPB proves itself to be a promising adjunct for multimodal analgesia.

Nonetheless while ESPB may show significant promise, it is prudent for practitioners to recognise early symptoms of Local Anaesthesia Systemic Toxicity (LAST). Like other interfascial plane blocks, ESPB are regarded as ‘volume’ blocks^44^ and local anaesthetic agents are injected in large volumes into a highly vascularised plane^45^ hence resulting in a significant risk of LAST. Studies^46^ have suggested that ESPB may result in LAST even when anaesthetic agents not administered at their maximum doses. Possible contributing factors include age, comorbids, and concurrent drug use^47^. To mitigate this, Shigeta *et al*.^48^ recommended the use of adjunctive epinephrine with local anaesthetics in patients with increased risk of LAST as epinephrine reduces the rate at which local anaesthetic agents diffuse into the plasma, delays systemic uptake, and decreases the toxic effect on vulnerable tissues, such as the myocardium and central nervous system (CNS). Yawata *et al*.^49^ also suggested that levobupivacaine could be associated with a higher risk of LAST in comparison to other agents such as ropivacaine. However, due to the relatively low incidence of LAST, further studies are needed to elucidate its risk factors and ways to reduce its occurrence.

This study is limited in terms of the number of available studies and high between-study heterogeneity which could impact the robustness of our findings. Due to the limited number of studies, subgroup analysis could not be performed to identify potential heterogeneity or compare ESPB in upper and lower abdominal surgeries which could potentially yield different pain experiences. While the various studies have ensured administration of anaesthetic agents into the erector spinae plane with the use of ultrasound guidance, there is unfortunately a lack of standardisation of the thoracic level as well as the concentration and volume of anaesthetic agents used. In addition, we recognise that the included studies had utilised two distinct pain assessment tools – VAS and NRS. Data on NRS was not included in our analysis as several RCTs employed a graphical representation of NRS and extrapolation of such data could compromise its reliability. Moreover, despite similarities between NRS and VAS, they are not parallel scores and direct conversions should not be made. As such, only VAS was analysed in our study. Lastly, other secondary outcomes such as time to bowel movement or length of hospital stay were not evaluated.

In conclusion, ESPB is a promising regional anaesthetic technique for perioperative analgesia after laparoscopic abdominal surgeries. Nonetheless, due to high levels of heterogeneity, larger samples, and high quality RCTs are required to further strengthen and verify our results.

## Ethical approval

Not applicable.

## Consent

Not applicable.

## Sources of funding

Not applicable.

## Author contribution

C.J.-Y.S.: study design, statistical analysis, interpretation of results, and writing of manuscript; S.-L.W.: interpretation of results and editing of manuscript; P.-S.A.-Y.: interpretation of results and editing of manuscript; S.-A.L.: interpretation of results and editing of manuscript; W.J.T.: interpretation of results and editing of manuscript; F.-J.F.: interpretation of results and editing of manuscript; K.J.H.: interpretation of results and editing of manuscript; D.J.K.L.: interpretation of results and editing of manuscript; F.H.K.: study design, interpretation of results, and editing of manuscript.

## Conflicts of interest disclosure

The authors declare that they have no financial conflict of interest with regard to the content of this report.

## Research registration unique identifying number (UIN)

This meta-analysis was registered at PROSPERO with No. CRD42023406896.

## Guarantor

Not applicable.

## Data availability statement

The authors confirm that the data supporting the findings of this study are available within the article.

## Provenance and peer review

Not commissioned, externally peer-reviewed.

## Presentation

Not applicable.

## Supplementary Material

SUPPLEMENTARY MATERIAL

## References

[1] Beverly A Kaye AD Ljungqvist O . Essential elements of multimodal analgesia in Enhanced Recovery After Surgery (ERAS) guidelines. Anesthesiol Clin 2017;35:e115–e143. 28526156 10.1016/j.anclin.2017.01.018

[2] Forero M Adhikary SD Lopez H . The erector spinae plane block. Reg Anesth Pain Med 2016;41:621–627. 27501016 10.1097/AAP.0000000000000451

[3] Gao Y Liu L Cui Y . Postoperative analgesia efficacy of erector spinae plane block in adult abdominal surgery: a systematic review and meta-analysis of randomized trials. Front Med (Lausanne) 2022;9:934866. 36267624 10.3389/fmed.2022.934866 PMC9578553

[4] Abd Ellatif SE Abdelnaby SM . Ultrasound guided erector spinae plane block versus quadratus lumborum block for postoperative analgesia in patient undergoing open nephrectomy: a randomized controlled study. Egypt J Anaesth 2021;37:123–134.

[5] Canitez A Kozanhan B Aksoy N . Effect of erector spinae plane block on the postoperative quality of recovery after laparoscopic cholecystectomy: a prospective double-blind study. Br J Anaesth 2021;127:629–635. 34340839 10.1016/j.bja.2021.06.030

[6] Koo CH Lee HT Na HS . Efficacy of erector spinae plane block for analgesia in thoracic surgery: a systematic review and meta-analysis. J Cardiothorac Vasc Anesth 2022;36:1387–1395. 34301447 10.1053/j.jvca.2021.06.029

[7] Sertcakacilar G Pektas Y Yildiz GO . Efficacy of ultrasound-guided erector spinae plane block versus paravertebral block for postoperative analgesia in single-port video-assisted thoracoscopic surgery: a retrospective study. Ann Palliat Med 2022;11:1981–1989. 35400156 10.21037/apm-22-75

[8] Gadsden J . The erector spinae plane block: the case of the elusive mechanism of action. Canad J Anesth 2021;68:288–292. 33403541 10.1007/s12630-020-01876-1

[9] Chin KJ El-Boghdadly K . Mechanisms of action of the erector spinae plane (ESP) block: a narrative review. Canad J Anesth 2021. 10.1007/s12630-021-02020-3 33978912

[10] Schwartzmann A Peng P Maciel MA . Mechanism of the erector spinae plane block: insights from a magnetic resonance imaging study. Canad J Anesth 2018;65:1165–1166. 30076575 10.1007/s12630-018-1187-y

[11] Jain K Jaiswal V Puri A . Erector spinae plane block: relatively new block on horizon with a wide spectrum of application - A case series. Indian J Anaesth 2018;62:809–813. 30443066 10.4103/ija.IJA_263_18 PMC6190410

[12] Lee THW Barrington MJ Tran TMN . Comparison of extent of sensory block following posterior and subcostal approaches to ultrasound-guided transversus abdominis plane block. Anaesth Intensive Care 2010;38:452–460. 20514952 10.1177/0310057X1003800307

[13] El-Boghdadly K Madjdpour C Chin KJ . Thoracic paravertebral blocks in abdominal surgery–a systematic review of randomized controlled trials. Br J Anaesth 2016;117:297–308. 27543524 10.1093/bja/aew269

[14] Block BM Liu SS Rowlingson AJ . Efficacy of postoperative epidural analgesia. JAMA 2003;290:2455. 14612482 10.1001/jama.290.18.2455

[15] Choi S Mahon P Awad IT . Neuraxial anesthesia and bladder dysfunction in the perioperative period: a systematic review. Canad J Anesth 2012;59:681–703. 22535232 10.1007/s12630-012-9717-5

[16] Page MJ McKenzie JE Bossuyt PM . The PRISMA 2020 statement: an updated guideline for reporting systematic reviews. Int J Surg 2021;88:105906. 33789826 10.1016/j.ijsu.2021.105906

[17] Shea BJ Reeves BC Wells G . AMSTAR 2: a critical appraisal tool for systematic reviews that include randomised or non-randomised studies of healthcare interventions, or both. BMJ 2017;358:j4008. 28935701 10.1136/bmj.j4008 PMC5833365

[18] Lillemoe K . Laparoscopic cholecystectomy as a “true” outpatient procedure: initial experience in 130 consecutive patients. J Gastrointest Surg 1999;3:44–49. 10457323 10.1016/s1091-255x(99)80007-9

[19] Arnold R Weissman DE . Calculating opioid dose conversions. Fast Facts and Concepts, 2nd ed. EPERC; 2005;36. http://www.eperc.mcw.edu/EPERC/FastFactsIndex/ff_036.htm

[20] Hozo SP Djulbegovic B Hozo I . Estimating the mean and variance from the median, range, and the size of a sample. BMC Med Res Methodol 2005;5:13. 15840177 10.1186/1471-2288-5-13 PMC1097734

[21] Choi JJ Chang YJ Lee D . Effect of erector spinae plane block on postoperative pain after laparoscopic colorectal surgery: a randomized controlled study. J Pers Med 2022;12:1717. 36294856 10.3390/jpm12101717 PMC9605267

[22] Ates I Disci E Yayik AM . Ultrasound-guided erector spinae plane block versus trocar site local anesthetic infiltration for laparoscopic colorectal resection: a prospective, randomized study. Ann Med Res 2022;29:62–67.

[23] Kim D Kim JM Choi GS . Ultrasound-guided erector spinae plane block for postoperative analgesia in laparoscopic liver resection: A prospective, randomised controlled, patient and observer-blinded study. Eur J Anaesthesiol 2021;38 (Suppl 2):S106–S112. 33653982 10.1097/EJA.0000000000001475

[24] Mostafa SF Abdelghany MS Abu Elyazed MM . Ultrasound‐guided erector spinae plane block in patients undergoing laparoscopic bariatric surgery: a prospective randomized controlled trial. Pain Pract 2021;21:445–453. 33295128 10.1111/papr.12975

[25] Qi-hong S Xu-yan Z Xu S . Comparison of ultrasound-guided erector spinae plane block and oblique subcostal transverse abdominis plane block for postoperative analgesia in elderly patients after laparoscopic colorectal Surgery: a Prospective randomized study. Pain Ther 2021;10:1709–1718. 34652717 10.1007/s40122-021-00329-x PMC8586115

[26] Rao Kadam V Ludbrook G van Wijk RM . A comparison of ultrasound guided bilateral single injection shot Erector Spinae Plane blocks versus wound infiltration for postoperative analgesia in laparoscopic assisted colonic surgery–a prospective randomised study. BMC Anesthesiol 2021;21:255. 34702183 10.1186/s12871-021-01474-8 PMC8547045

[27] Kang R Chin KJ Kim GS . Bilateral continuous erector spinae plane block using a programmed intermittent bolus regimen versus intrathecal morphine for postoperative analgesia in living donor laparoscopic hepatectomy: a randomized controlled trial. J Clin Anesth 2021;75:110479–110479. 34455152 10.1016/j.jclinane.2021.110479

[28] Warner M Yeap YL Rigueiro G . Erector spinae plane block versus transversus abdominis plane block in laparoscopic hysterectomy. Pain Manag 2022;12:907–916. 36214314 10.2217/pmt-2022-0037

[29] Xu ZZ Li X Chen B-L . A randomised controlled trial of the non‐inferiority of erector spinae plane block vs. thoracic paravertebral block for laparoscopic nephro‐ureterectomy. Anaesthesia 2023;78:442–448. 36599621 10.1111/anae.15959

[30] Viderman D Aubakirova M Abdildin YG . Erector spinae plane block in abdominal surgery: a meta-analysis. Front Med 2022;9:812531. 10.3389/fmed.2022.812531 PMC8904394 35280917

[31] Kemp J . Unreliability of the visual analog scale in experimental pain assessment: a sensitivity and evoked potentials study. Pain Physician 2012;5;15:E693–E699. 22996863

[32] Rafi AN . Abdominal field block: a new approach via the lumbar triangle. Anaesthesia 2001;56:1024–1026. 10.1046/j.1365-2044.2001.02279-40.x 11576144

[33] Pawa A King C Thang C . Erector spinae plane block: the ultimate “plan A” block? Br J Anaesth 2023;130:497–502. 36775671 10.1016/j.bja.2023.01.012

[34] White L Riley B Davis K . Safety of continuous erector spinae catheters in chest trauma: a retrospective cohort study. Anesth Anal 2021;133:1296–1302. 10.1213/ANE.0000000000005730 34473654

[35] Luff D Moosa F Sadavarte N . 136 Which blocks can you do? An assessment of anaesthetic trainee confidence performing common peripheral nerve blocks. Peripheral Nerve Blocks Regional Anesthesia & Pain Medicine 2021;70:A71.

[36] Cassai A Geraldini F Carere A . Complications rate estimation after thoracic erector spinae plane block. J Cardiothorac Vasc Anesth 2021;35:3142–3143. 33731296 10.1053/j.jvca.2021.02.043

[37] Fang B Wang Z Huang X . Ultrasound-guided preoperative single-dose erector spinae plane block provides comparable analgesia to thoracic paravertebral block following thoracotomy: a single center randomized controlled double-blind study. Ann Transl Med 2019;7:174. 31168455 10.21037/atm.2019.03.53 PMC6526263

[38] El Ghamry M Amer A . Role of erector spinae plane block versus paravertebral block in pain control after modified radical mastectomy. A prospective randomised trial. Indian J Anaesth 2019;63:1008. 31879425 10.4103/ija.IJA_310_19 PMC6921308

[39] Sen S Morrison B O’Rourke K . Analgesia for enhanced recovery after surgery in laparoscopic surgery. Dig Med Res 2019;2:25.

[40] Zubair M Adil Khan M Khan MNA . Comparison of continuous thoracic epidural with erector spinae block for postoperative analgesia in adult living donor hepatectomy. Cureus 2022;14:e23151. 35444875 10.7759/cureus.23151 PMC9010007

[41] Luis-Navarro JC Seda-Guzmán M Luis-Moreno C . Erector spinae plane block in abdominal surgery: case series. Indian J Anaesth 2018;62:549–554. 30078859 10.4103/ija.IJA_57_18 PMC6053882

[42] Borzellino G Francis NK Chapuis O . Role of epidural analgesia within an eras program after laparoscopic colorectal surgery: a review and meta-analysis of randomised controlled studies Surg Res Pract 2016;2016:1–9. 10.1155/2016/7543684 PMC5013204 27642630

[43] Hermanides J Hollmann MW Stevens MF . Failed epidural: causes and management. Br J Anaesth 2012;109:144–154. 22735301 10.1093/bja/aes214

[44] Hamilton DL . Local anesthetic systemic toxicity following erector spinae plane block: sometimes less is more. Korean J Anesthesiol 2021;74:361–362. 33198429 10.4097/kja.20596 PMC8342833

[45] Yue BY le Roux CM Corlett R . The arterial supply of the cervical and thoracic spinal muscles and overlying skin: anatomical study with implications for surgical wound complications. Clin Anat 2013;26:584–591. 22887027 10.1002/ca.22139

[46] Lee JY Kim HT Won JM . A rare case of Euphoria caused by lidocaine after an erector spinae plane block: a case report. J Pain Res 2020;13:2329–2332. 33061547 10.2147/JPR.S271535 PMC7519830

[47] Heppolette CAA Brunnen D Bampoe S . Clinical pharmacokinetics and pharmacodynamics of levobupivacaine. Clin Pharmacokinet 2020;59:715–745. 32034727 10.1007/s40262-020-00868-0

[48] Shigeta H Yasumura R Kotake Y . Comparison of plasma levobupivacaine concentrations with and without epinephrine following erector spinae plane block for breast cancer surgery: a randomized controlled trial. BMC Anesthesiol 2022;22:86. 35350983 10.1186/s12871-022-01632-6 PMC8966335

[49] Yawata S Imamachi N Sakura S . Local anesthetic systemic toxicity of levobupivacaine in erector spinae plane block. Korean J Anesthesiol 2021;74:271–272. 33099297 10.4097/kja.20560 PMC8175880

