# Supplementary material for: Analgesia efficacy of erector spinae plane block in laparoscopic abdominal surgeries: a systemic review and meta-analysis
Source: Int J Surg. 2024 Apr 3;110(7):4393–401. doi: 10.1097/JS9.0000000000001421 (PMC11254305; doi:10.1097/JS9.0000000000001421)
Supplement: SUPPLEMENTARY MATERIAL [file js9-110-4393-s001.docx]

**Supporting Information Table 1**

Sensitivity Analysis

| Post-operative opioid consumption | | |  |  |  |  |  |  |
| --- | --- | --- | --- | --- | --- | --- | --- | --- |
| Study Removed | | Hedges's g Effect Size | Lower Limit | Upper Limit | p-value |  |  |  |
| Mostafa S.F et al 2021 | | 0.812 | 0.160 | 1.463 | 0.015 |  |  |  |
| Qi Hong S et al 2021 | | 0.964 | 0.235 | 1.692 | 0.010 |  |  |  |
| Rao Kadam V et al 2021 | | 1.115 | 0.441 | 1.789 | 0.001 |  |  |  |
| Ryung A Kang et al 2021 | | 1.200 | 0.637 | 1.763 | 0.000 |  |  |  |
| Kim D et al 2021 | | 1.082 | 0.378 | 1.787 | 0.003 |  |  |  |
| I Ates et al 2022 | | 0.971 | 0.250 | 1.691 | 0.008 |  |  |  |
| Choi JJ et al 2022 | | 0.894 | 0.193 | 1.596 | 0.012 |  |  |  |
| Warner M et al 2022 | | 0.91 | 0.196 | 1.626 | 0.013 |  |  |  |
| Xu ZZ 2023 | | 0.891 | 0.184 | 1.597 | 0.013 |  |  |  |
|  | | 0.992 | 0.335 | 1.629 | 0.003 |  |  |  |
|  | |  |  |  |  |  |  |  |
| Intra-operative opioid consumption | |  |  |  |  |  |  |  |
| Study Removed | | Hedges's g Effect Size | Lower Limit | Upper Limit | p-value |  |  |  |
| Mostafa S.F et al 2021 | | 0.406 | -0.230 | 1.043 | 0.211 |  |  |  |
| Rao Kadam V et al 2021 | | 1.465 | 0.023 | 2.907 | 0.047 |  |  |  |
| Choi JJ et al 2022 | | 1.140 | -0.938 | 3.219 | 0.282 |  |  |  |
| Overall | | 0.998 | -0.158 | 2.154 | 0.091 |  |  |  |
|  | |  |  |  |  |  |  |  |
| Post-operative Pain Score (VAS) | |  |  |  |  |  |  |  |
| Study Removed | | Hedges's g Effect Size | Lower Limit | Upper Limit | p-value |  |  |  |
| Qi Hong S et al 2021 0.461 | | | -0.564 | 1.486 | 0.378 |  |  |  |
| Mostafa S.F et al 2021 | | 0.438 | -0.555 | 1.432 | 0.387 |  |  |  |
| I Ates et al 2022 | | 0.539 | -0.530 | 1.607 | 0.323 |  |  |  |
| Warner M et al 2022 | | 0.989 | 0.674 | 1.304 | 0.000 |  |  |  |
| Overall | | 0.602 | -0.197 | 1.401 | 0.140 |  |  |  |
|  | |  |  |  |  |  |  |  |
| Post-operative Nausea | |  |  |  |  |  |  |  |
| Study Removed | | Odds Ratio (OR) | Lower Limit | Upper Limit | p-value |  |  |  |
| Qi Hong S et al 2021 | | 0.278 | 0.053 | 1.459 | 0.130 |  |  |  |
| Ryung A Kang et al 2021 | | 0.398 | 0.133 | 1.171 | 0.094 |  |  |  |
| I Ates et al 2022 | | 0.244 | 0.048 | 1.239 | 0.089 |  |  |  |
| Warner M et al 2022 | | 0.148 | 0.064 | 0.347 | 0.000 |  |  |  |
| Overall | | 0.250 | 0.078 | 0.801 | 0.020 |  |  |  |
|  | |  |  |  |  |  |  |  |
| Post-operative Vomiting | |  |  |  |  |  |  |  |
| Study Removed | | Odds Ratio (OR) | Lower Limit | Upper Limit | p-value |  |  |  |
| Qi Hong S et al 2021 | | 0.223 | 0.076 | 0.650 | 0.006 |  |  |  |
| Ryung A Kang et al 2021 | | 0.317 | 0.091 | 1.105 | 0.071 |  |  |  |
| I Ates et al 2022 | | 0.148 | 0.051 | 0.426 | 0.000 |  |  |  |
| Overall | | 0.209 | 0.088 | 0.499 | 0.000 |  |  |  |
|  | |  |  |  |  |  |  |  |
| Laparoscopic Colorectal Surgery - Post-operative Opioid Consumption | |  |  |  |  |  |  |  |
| Study Removed | | Hedges's g Effect Size | Lower Limit | Upper Limit | p-value |  |  |  |
| Qi Hong S et al 2021 0.185 -0.142 0.512 | | | | | 0.268 |  |  | 0.1 |
| Rao Kadam V et al 2021 | | 0.609 | 0.045 | 1.174 | 0.034 |  |  |  |
| Choi JJ et al 2022 | | 0.525 | -0.181 | 1.232 | 0.145 |  |  |  |
| I Ates et al 2022 | | 0.409 | -0.298 | 1.116 | 0.257 |  |  |  |
| Overall | | 0.434 | -0.084 | 0.953 | 0.101 |  |  |  |
|  | |  |  |  |  |  |  |  |
| Laparoscopic Colorectal Surgery - Intra-operative Opioid Consumption | |  |  |  |  |  |  |  |
| Study Removed | | Hedges's g Effect Size | Lower Limit | Upper Limit | p-value |  |  |  |
| Rao Kadam V et al 2021 0.741 0.220 1.262 | | | | | 0.005 |  |  | 0.005 |
| Choi JJ et al 2022 | | 0.091 | -0.383 | 0.565 | 0.707 |  |  |  |
| Overall | | 0.406 | -0.230 | 1.043 | 0.211 |  |  |  |
|  |  | |  |  |  |  |  |  |
| Laparoscopic Colorectal - Post-Operative Pain Score (VAS) |  | |  |  |  |  |  |  |
| Study Removed | | Hedges's g Effect Size | Lower Limit | Upper Limit | p-value |  |  |  |
| Qi Hong S et al 2021 0.807 0.227 | | | | 1.386 | 0.006 |  |  |  |
| I Ates et al 2022 | | 1.032 | 0.508 | 1.556 | 0 |  |  |  |
| Overall | | 0.931 | 0.542 | 1.319 | 0 |  |  |  |
|  | |  |  |  |  |  |  |  |
| Post-Operative Opioid Consumption (ESPB vs Sham Block) | |  |  |  |  |  |  |  |
| Study Removed | | Hedges's g Effect Size | Lower Limit | Upper Limit | p-value |  |  |  |
| Choi JJ 2022 | | 1.290 | -0.877 | 3.458 | 0.243 |  |  |  |
| Kim D 2021 | | 2.016 | 1.292 | 2.739 | 0.000 |  |  |  |
| Mostafa S F 2021 | | 0.926 | -0.517 | 2.369 | 0.208 |  |  |  |
| Overall | | 1.410 | 0.104 | 2.716 | 0.034 |  |  |  |

| Post-Operative Opioid  Consumption (ESPB vs TAPB) |  |  |  |  |
| --- | --- | --- | --- | --- |
| Study Removed | Hedges's g Effect Size | Lower  Limit | Upper  Limit | p-value |
| Qi Hong 2021 | 1.554 | 1.037 | 2.070 | 0.000 |
| Warner M 2022 | 1.137 | 0.606 | 1.668 | 0.000 |
| Overall | 1.350 | 0.942 | 1.758 | 0.000 |
| Post-Operative Opioid  Consumption (ESPB vs Wound Infiltration) |  |  |  |  |
| Study Removed | Hedges's g Effect Size | Lower  Limit | Upper  Limit | p-value |
| I Ates 2022 | -0.066 | -0.547 | 0.415 | 0.789 |
| Rao Kadam V 2021 | 1.080 | 0.483 | 1.677 | 0.000 |
| Overall | 0.493 | -0.630 | 1.616 | 0.389 |
